# A Novel Evolution-Based Method for Detecting Gene-Gene Interactions

**DOI:** 10.1371/journal.pone.0026435

**Published:** 2011-10-25

**Authors:** Shaoqi Rao, Manqiong Yuan, Xiaoyu Zuo, Weiyang Su, Fan Zhang, Ke Huang, Meihua Lin, Yuanlin Ding

**Affiliations:** 1 Department of Medical Statistics and Epidemiology, School of Public Health, Guangdong Medical College, Dongguan, Guangdong, China; 2 Department of Statistical Sciences, School of Mathematics and Computational Science, Sun Yat-Sen University, Guangzhou, Guangdong, China; 3 Department of Medical Statistics and Epidemiology, School of Public Health, Sun Yat-Sen University, Guangzhou, Guangdong, China; 4 Institute of Blood Transfusion, Guangzhou Blood Center, Guangzhou, Guangdong, China; University of Texas School of Public Health, United States of America

## Abstract

**Background:**

The rapid advance in large-scale SNP-chip technologies offers us great opportunities in elucidating the genetic basis of complex diseases. Methods for large-scale interactions analysis have been under development from several sources. Due to several difficult issues (e.g., sparseness of data in high dimensions and low replication or validation rate), development of fast, powerful and robust methods for detecting various forms of gene-gene interactions continues to be a challenging task.

**Methodology/Principal Findings:**

In this article, we have developed an evolution-based method to search for genome-wide epistasis in a case-control design. From an evolutionary perspective, we view that human diseases originate from ancient mutations and consider that the underlying genetic variants play a role in differentiating human population into the healthy and the diseased. Based on this concept, traditional evolutionary measure, fixation index (*Fst*) for two unlinked loci, which measures the genetic distance between populations, should be able to reveal the responsible genetic interplays for disease traits. To validate our proposal, we first investigated the theoretical distribution of *Fst* by using extensive simulations. Then, we explored its power for detecting gene-gene interactions via SNP markers, and compared it with the conventional Pearson Chi-square test, mutual information based test and linkage disequilibrium based test under several disease models. The proposed evolution-based method outperformed these compared methods in dominant and additive models, no matter what the disease allele frequencies were. However, its performance was relatively poor in a recessive model. Finally, we applied the proposed evolution-based method to analysis of a published dataset. Our results showed that the *P* value of the *Fst* -based statistic is smaller than those obtained by the LD-based statistic or Poisson regression models.

**Conclusions/Significance:**

With rapidly growing large-scale genetic association studies, the proposed evolution-based method can be a promising tool in the identification of epistatic effects.

## Introduction

Most complex diseases have a sophisticated molecular etiology, typically involving multiple genes and their non-linear interactions. There is a growing consensus that gene-gene interaction assay is an important avenue for the discovery of genetic exposures related to complex disease. In genome-wide association studies (GWAS), majority of genes may be with only small effects that could hardly be detected by current single-locus methodology. However, it is reasonable to believe that the combination of some of these small effect genes could create more effect than their summation in a simple way [1]. For example, the odds ratio of the interaction effect of two genes may be much larger than their combined (sum or product) effect [2], [3]. Therefore, the analysis considering gene-gene interaction instead of single genes has become inevitable.

In the past decade, several statistical methods have been developed for detecting gene-gene interaction. For examples, standard logistic regression is the method most commonly used to test multiplicative interaction effects. Genetic algorithm (GA), which based on the principle of the survival competition, mainly aims at finding out the fittest gene combination according to a specific fitness function. And multifactor dimensionality reduction (MDR) [4] is a nonparametric method for detecting and characterizing high-order gene-gene interaction in case-control studies with relatively small samples. Moreover, classification tree model [5] has been seen in detecting gene-gene interaction. It creates a binary tree and each path of the tree can be treated as a combination of some related genes.

However, several limitations exist in these currently available methods. Most parametric-statistical methods, e.g. standard logistic regression, are impractical for dealing with high-throughput data. That because when high-order interactions are considered, there are many empty cells in the contingency table [6]. And some obvious deficiencies also exist in some nonparametric methods. For example, the solution of GA may just be a local optimum rather than the global optimum and its convergence rate might be relatively small, causing time-consuming; the results of MDR, as Moore et al. [7] pointed out, were hard to be interpreted because it ignored the interaction effects from the viewpoint of biology or genetics; and the establishment of classification tree would strongly depend on or influenced by the effect of a parent node, that is, when the parent node changed, the tree may be largely different, thus lacking robustness to a feature(s) with a strong main effect.

To overcome these limitations, we propose a novel evolution perspective to trace the origins of diseases. Most recently, a new field, called evolutionary medicine [8]–[10], begins to emerge. It applies modern evolutionary theory to understand health and disease, and provides a complementary scientific approach to the present mechanistic explanations of human disease that dominate medical science. As Nesse et al. [11] pointed out, all biological traits need two kinds of explanation, both proximate and evolutionary. The proximate explanation for a disease describes what is wrong in the bodily mechanism of individuals affected by it. An evolutionary explanation tells why we are all the same in ways that leave us vulnerable to disease. While traditionally viewed that natural selection could explain only health rather than disease, arguments have been raised that natural selection maximizes the reproductive success of genes or gene combinations [12], [13]. In other words, those genes or gene-gene interaction that confer individuals' superior reproduction will likely become more common, even if they caused health problems or disease [14]. There fore, to better probe genetic basis for human health-related problems, there is a growing demand for incorporating both proximate and evolutionary explanation [15]. Scientists in the field of evolutionary medicine conclude some selected principles which provide a foundation for considering disease in an evolutionary context. “Disease is inevitable because of the way that organisms are shaped by evolution” and “Disease are not products of natural selection, but most of the vulnerabilities that lead to disease are shaped by the process of natural selection” [12] are two of these selected principles of evolutionary medicine. Therefore, it is reasonable to hypothesize that the origin and progression of disease resulted from evolution.

Based on the above perspective, we view that human diseases originate from ancient mutations and consider that the underlying genetic variants play a role in differentiating human population, into the healthy and the diseased. By this reasoning, traditional evolutionary measure, fixation index (*Fst*) for two unlinked loci, which measures the genetic distance between populations, should be able to reveal the responsible genetic interplays for a disease trait, and also provides valuable insights into the evolutionary process of complex disease [16]. *Fst* is a special case of F-statistics [17], a concept developed in 1920s by Sewall Wright [18]. It is mainly a measure of population differentiation and genetic distance. And it can also reflect the correlation that gametes or haplotypes chosen randomly from within the same subpopulation relative to the entire population [16]. When the frequencies of gametes or haplotypes differ between the two subpopulations, it can be interpreted as evidence for relationship between the markers and disease-related genes. This in turn suggests that we can apply two-loci *Fst* as a measure of gene-gene interaction that is related to the disease.

The main purpose of this article was to develop a statistic with high power for detection of gene-gene interaction between two unlinked loci. To accomplish this, we first described how gene-gene interaction could impact the value of *Fst*. We then studied the theoretical distribution of *Fst* under the null hypothesis that two loci are absent of interaction between each other, followed by validation of the null distribution by extensive simulations. We evaluated the statistical power of the proposed evolution-based approach to detecting gene-gene interaction under several disease models and compared it with several alternative methods. We found that the proposed evolution-based method outperformed these alternative methods in dominant and additive models, while it performed relatively poor in a recessive model. To further evaluate the performance of the proposed method, we also applied it to a real example about the sickle cell disease and malaria. Our results showed that the *P* value of the *F*st -based statistic was smaller than those obtained by the LD-based statistic or logistic regression models. Finally, we concluded this report with a discussion of the advantages and potential limitations of our proposed method.

## Methods

### Two-loci Fixation Index (*Fst*)

Sewall Wright [18] introduced *Fst* as one of the three interrelated parameters, *Fis*, *Fit* and *Fst*, to describe the genetic structure of diploid populations. As mentioned above, it measures the correlation between gametes or haplotypes chosen randomly from within the same subpopulation and those from the entire population. The concept of *Fst* arises from evolutionary theory. From the genetic viewpoint, evolution can be defined as a change from generation to generation in the frequencies of gametes within a population that shares a common gene pool [19], [20]. It occurs when there are changes in the frequencies of gametes or haplotypes within a population of interbreeding organism. In this article, the disease status was regarded as a classification feature, according to which we could separate the population into case group and control group. Based on the abovementioned perspective about the origins of disease, case group and control group can be treated as two subpopulations diverged from a common healthy ancestor. Naturally, the two subpopulations have a great mount of common characters, while some different genetic factors do exist if they are responsible for disease status.

For a diploid population, let *A* and *a* be the two alleles at the first disease locus, with observed frequencies *p_A_* and *p_a_*, respectively. Let *B* and *b* be the two alleles at the second disease locus, with observed frequencies *p_B_* and *p_b_*, respectively. Each locus has three genotypes coded as 0, 1 and 2. Let random variable *X_A_* takes 1 for allele *A* and 0 for allele *a*. *X_B_* is similarly defined. We then define a random bivariable **X** = (*X_A_*, *X_B_*). **X** can take four possible vectors (1,1), (1,0), (0,1) and (0,0), which represent two-loci gametes *AB*, *Ab*, *aB*, and *ab*, respectively. Suppose our research population is a large random-mating population, therefore **X** has a multinomial distribution with index one and parameter **h** = (*h_AB_*, *h_Ab_*, *h_aB_*, *h_ab_*), denoted by *multinomial* (1, **h**), where **h** are the population gametes frequencies of *AB*, *Ab*, *aB*, and *ab*, respectively. According to our definitions, both *X_A_* and *X_B_* obey a Bernoulli distribution with mean *μ_A_* and *μ_B_*, respectively, where *μ_A_* and *μ_B_* are the population frequencies of the two disease alleles which equal to *h_AB_*+*h_Ab_* and *h_AB_*+*h_aB_*, respectively. From the properties of the Bernoulli distribution, we have the unbiased estimates for means **μ** = (*μ_A_*, *μ_B_*), variances **σ^2^** = (*σ* ^2^ *_A_*, *σ* ^2^ *_B_*), and covariance of *X_A_* and *X_B_* (*σ* ^2^ *_AB_*), as follows:
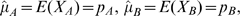


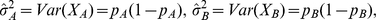


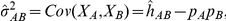
where 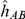 is the maximum likelihood estimation of the frequency of gamete *AB*. In most studies, the raw data are genotypes and hence we can not compute 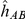 directly by the proportion of gametes *AB*. As a result, we have first to estimate 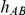 from genotype data. In our study, we employ an EM algorithm [21] to search for the numerical value of maximum likelihood estimation of *h_AB_*. Denote 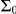, 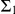 and 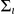 as the estimates of covariance matrix of **X** = (*X_A_*, *X_B_*) for control group, case group, and the entire population, respectively. We have
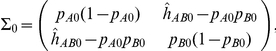


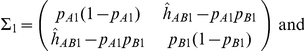


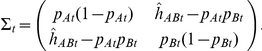



To derive the *Fst* statistic, we assume: (1) the observed gametes **x** *_i_*_1_, **x** *_i_*_2_, … , **x** *_ini_* are independently and identically sampled from a multinomial distributed population with *multinomial* (1, **h** *_i_*), *i* = 0,1, where 0 stands for control group and 1 for case group, respectively, and *n_i_* is the size of sampled gametes in *i* ^th^ group; (2) the null hypothesis of our test is that case group and control group have equal frequencies of gametes, which could be formulated as
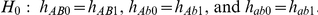



It can be seen that this hypothesis formulation is equivalent to the one set in multivariate analysis of variance (MANOVA). In terms of multivariate analysis of variance, **h** *_i_*, the *i*-th population mean haplotypes frequencies, can be decomposed into the overall mean component (**h**) and a component due to the specific population effect (**a** *_i_*):
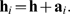



Hence, the null hypothesis can be alternatively written as *H*_0_: **a**_0_ = **a**_1_ = **0**. The vector of observation **x** *_ij_* can be described by a linear model [22]:
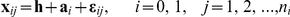
where **ε** ***_ij_*** is the error term vector that accounts for the uncertainties in **x** *_ij_*. As in the general MANOVA model, the following constraint applies:
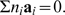



Given the above assumptions and for large sample size association studies, we can use multivariate analysis of variance (MANOVA) technique to test the null hypothesis that the frequencies of the haplotypes are the same in case group and control group. It should be noted that the independence assumption for MANOVA is not met in the SNP-based association studies because the bivariable **X** for two-loci gametes only take four discrete values. This violation has an impact on the sampling covariance matrix of **X**. However, we can prove that its asymptotic matrix is equivalent to the formulations for normally distributed variables, when the sample size of gamete *n* is large (see Text S1 and supplementary Figure S1 for details).

Now, we apply the definition of *Fst* for multiple loci:
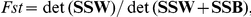
where **SSW** and **SSB** are the sum of square and cross-product matrices of ***X*** = (*X_A_*, *X_B_*) within and between populations, respectively. For the scenario of two loci, we have:
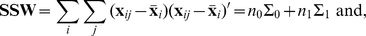


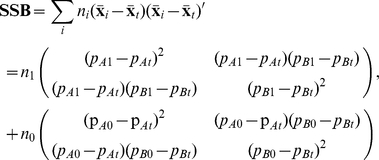
where *n* is the sample size of gamete, and *p* is the observed allele frequency. The subscripts refer to the two subpopulations (0 for control and 1 for case) or total population (t), as mentioned above. The degree of freedom of **SSW** and **SSB** are *n*_0_+*n*_1_−2 and 1, respectively.

For large sample size, we know that under the null hypothesis *Fst* approximately follows a Wilks' lambda distribution [23], Λ(*k,n−m,m−1*), where *k* is the dimension of **X**, *n* is the total sample size of gametes, and *m* is the number of groups. For diploid species and two loci, *k* = 2, *m* = 2 and *n* is two times total number of subjects, because one subject has two gametes or haplotypes.

When the sample size *n* is large and the null hypothesis *H*_0_ is true, *Fst* can be transformed (mathematically adjusted) to a statistic which has approximately an *F* distribution [24]. The transformation is as follows [23]:
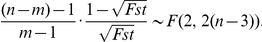



Consequently, we reject the null hypothesis *H*_0_ at significance *α* if
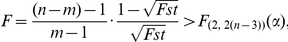
where *F*_(2, 2(*n*−3))_(*α*) is the upper (100*α*)th percentile of the *F* distribution with degree of freedom 2 and 2(*n*−3).

### Mutual Information (*MI*) and Linkage Disequilibrium Measure (*r^2^*)

For comparison, we also briefly describe two alternative methods for detecting gene-gene interaction, the mutual information (*MI*) based method and the linkage disequilibrium based method. Zhao et al. [25] proved that the entropy, a basic concept of *MI*, in context of genetic association studies, can reflect the association strength between the marker and the studied disease by its difference between the affected and unaffected individuals. Define SNP pair as a random variable (*S*) which has nine genotypes: *AABB*, *AaBB*, *aaBB*, *AABb*, *AaBb*, *aaBb*, *AAbb*, *Aabb*, and *aabb*. And disease status (*Y*) is another random variable with two statuses (case and control). Li et al. [26] applied the following definition of *MI* to test the association between SNP and the disease:
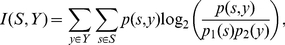
(1)where *p*(*s*, *y*) is the joint probability distribution function of *S* and *Y*, *p_1_*(*s*) and *p_2_*(*y*) are the marginal probability distribution of *S* and *Y*, respectively. If *S* and *Y* are independent, we have 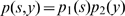, from which we can easily see that *I*(*S*, *Y*) equals to zero. And according to the definition above, the larger the value of mutual information is, the closer correlation the SNP pair and the disease have. Brillinger [27] pointed out that in the case of Li's definition with large sample size *n*, *MI* would approximately follows 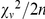 distribution, where *n* is the sample size and *ν* equals (*I*−1)(*J*−1), *I* and *J* are the numbers of value that *S* and *Y* could take, respectively. Here, *I* = 9 and *J* = 2 and therefore *ν* is 8.

The definition of the linkage disequilibrium measure *r* ^2^ is
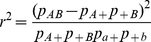
(2)where *p_AB_*, *p_Ab_*, *p_aB_*, and *p_ab_* are the frequencies of gametes *AB*, *Ab*, *aB* and *ab*, respectively. And the marginal probabilities are 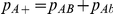, 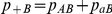, 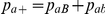, and 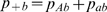. As well known, when two loci are in linkage equilibrium, the distribution of *Nr* ^2^ follows *χ* ^2^_(1)_, where *N* is the sample size of gamete data. Here, we should emphasize that although one genotype sample could create two gametes, for two loci with two alleles each, once one gamete is clear, the other can be completely determined. For example, consider the genotype *AaBb*, and it can be created by two kinds of gametes combinations: *AB* and *ab*, or *Ab* and *aB*. Given one of the gametes, such as *AB*, we can completely determine that the other gamete is *ab*. So, although *N* genotype samples can create *2N* haplotypes, *Nr* ^2^ not 2*Nr* ^2^ obeys *χ* ^2^_(1)_. Zhao et al proposed an improved LD-based statistic by comparing the difference of *r* ^2^ between two groups (case and control) to test the gene-gene interaction. In this study, we have performed a power comparison between the simple LD-based method and the improved LD-based statistic proposed by Zhao et al. [28]. Only subtle difference was observed between the two methods in terms of statistical power curves. Therefore, results from the former are shown in this article.

## Results

### Null Distribution of *Fst*

**Figure 1:**
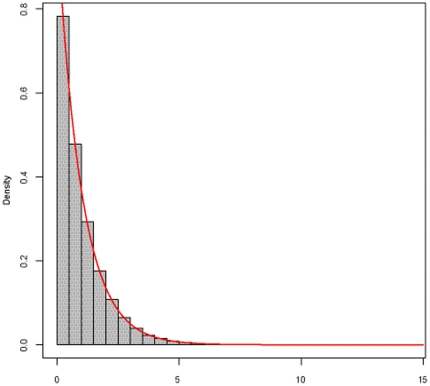
Frequency histogram of the *Fst* based statistic based on 10,000 simulations, compared with *F*(2, 3994). The gray bar denotes the frequency histogram of the *Fst* based statistic, corresponding to the null hypothesis that two SNPs are of no interaction. The red line is the density curve of the theoretical one.

**Figure 2:**
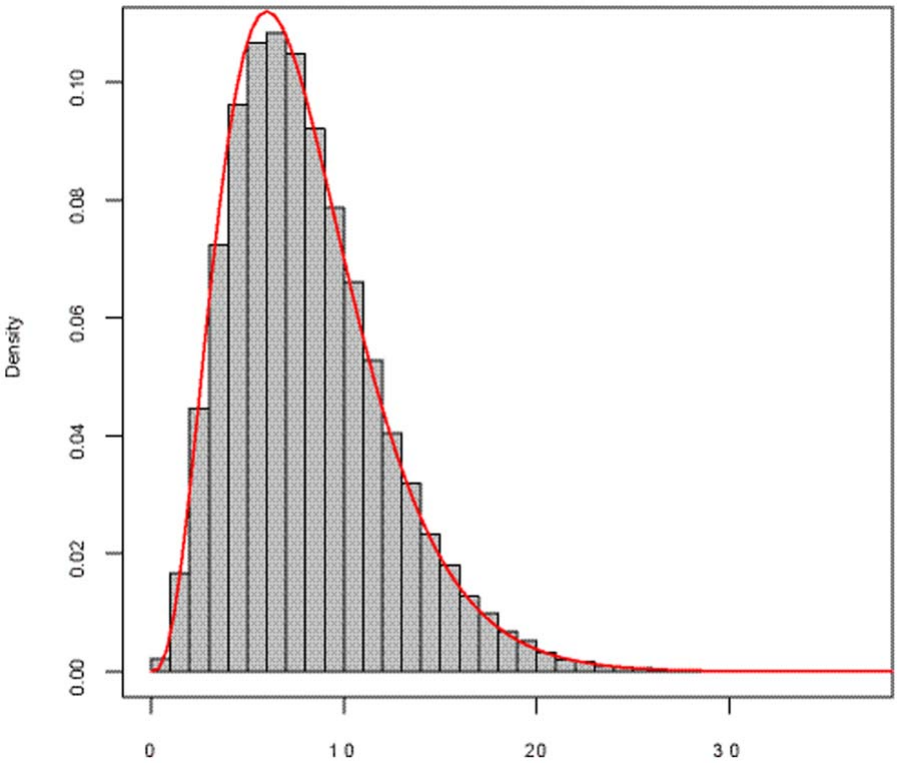
Frequency histogram of 2000×*MI* based on 10,000 simulations, compared with χ^2^(8). The gray bar denotes the frequency histogram of 2000×*MI*, corresponding to the null hypothesis that two SNPs are of no interaction. The red line is the density curve of χ^2^(8).

**Figure 3:**
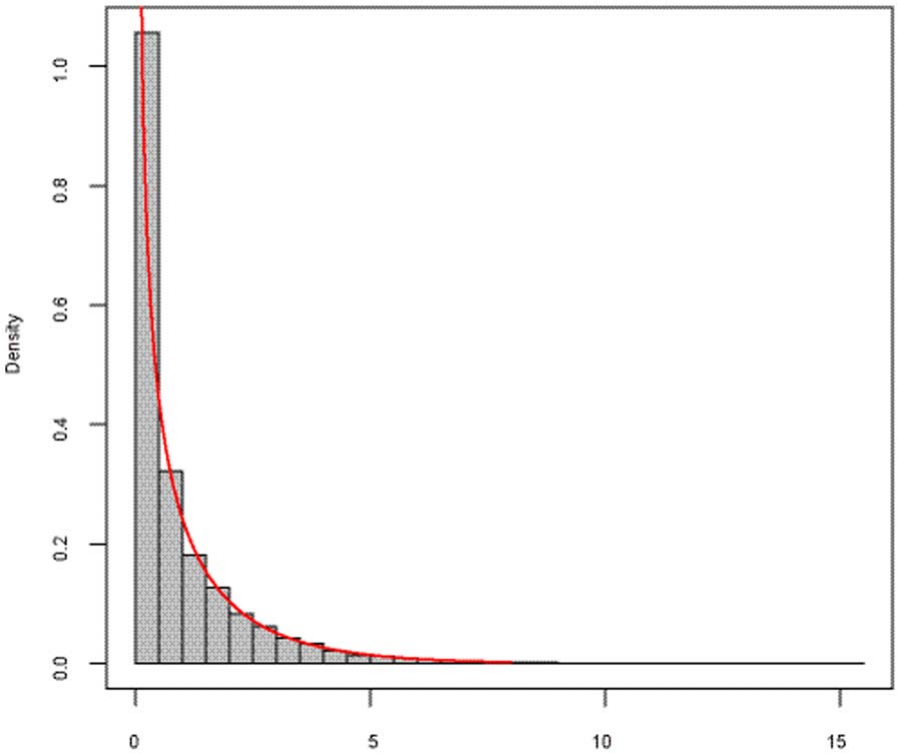
Frequency histogram of the *LD* based statistic based on 10,000 simulations, compared with χ^2^(1). The gray bar denotes the frequency histogram of the LD based statistic, corresponding to the null hypothesis that two SNPs are of no interaction. The red line is the density curve of the theoretical one.

As mentioned above, when the sample size is large, the transformation of *Fst* under the null hypothesis asymptotically approximates the *F* distribution. To validate this statement, we performed a large number of simulations using Matlab software. First, we randomly generated two independent minor allele frequencies (MAF) for two loci based on a uniform distribution ranging from 0.1 to 0.4. We then generated 1000 individuals with independent genotypes at two loci, coded as 0, 1 and 2, which were conformable to the Hardy-Weinberg equilibrium. Finally, we randomly selected 500 individuals as cases and the others as controls. As a result, we created a dataset with 1000 individuals and each had three parts, disease status and genotypes of the two loci, respectively. A total of 10,000 simulations were conducted to obtain the empirical distribution of *Fst*. Here, *n* = 2000, *p* = 2, and *m* = 2. So, the abovementioned transformation of *Fst* is asymptotically distributed as:
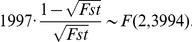



Figure 1 gives the frequency histogram of the 10,000 *F* values, and the density curve of *F*(2, 3994). It can be seen that the empirical distribution approximates the theoretical distribution well. We further evaluated the goodness-of-fit between the empirical one and theoretical one by using Kolmogorov-Smirnov test, demonstrating that no significant difference between the two distributions (p-value = 0.2391) was observed. Meantime, we investigated the frequency histograms of simulated *MI* values and *r^2^* values, and both empirical distributions appear to be in good agreement with their corresponding asymptotic distributions (see Figures 2 and 3).

### Power Evaluation

To evaluate the performance of *Fst* for detecting gene-gene interaction, we compared its power to those of Pearson's Chi-square test, *MI* and *r^2^*. The simulation software genomeSIMLA, a forward time simulation for genetic data [29], was used to generate case-control samples. We first generated two chromosomes, one containing 5 SNPs and the other containing 8 SNPs. Only two (g1 and g2) of these 13 SNPs were associated with the binary disease phenotype. The recombination fractions between SNP loci were randomly selected between 0.000001 and 0.00001. Three interaction models were investigated: dominant×dominant, additive×additive and recessive×recessive. For each model, 1000 datasets were simulated. Each dataset contained 1000 individuals (500 cases and 500 controls) and each individual had data for disease status and 13 genotypes, coded as 0,1and 2. The prevalence of the simulated disease was assumed to be 1%.

For a specific interaction model, two different sets of disease allele frequencies were considered: (1) *f*(*A*) = 0.2, *f*(*B*) = 0.4; (2) *f*(*A*) = 0.3, *f*(*B*) = 0.8, where *A* and *B* were disease alleles at the two loci. To explore the power of *Fst* for detecting gene-gene interaction under different parameter settings, 11 different interaction effects (g1×g2) were simulated, corresponding to ln(RR_g1×g2_) = 0, 0.05, 0.1, 0.2, 0.3, 0.5, 0.6, 0.65, 0.8, and 1.3, respectively, where RR_g1×g2_ was the relative risk of g1×g2. First, we computed the value of *Fst*, and then the derived *F* statistic. Significance was claimed when the observed *F* statistic is larger than the theoretical critical value (95% percentile of *F*(2,3994). The power of *Fst* for detecting gene interaction was defined as the proportion of significance in all the tests for 1000 simulated datasets.

**Figure 4:**
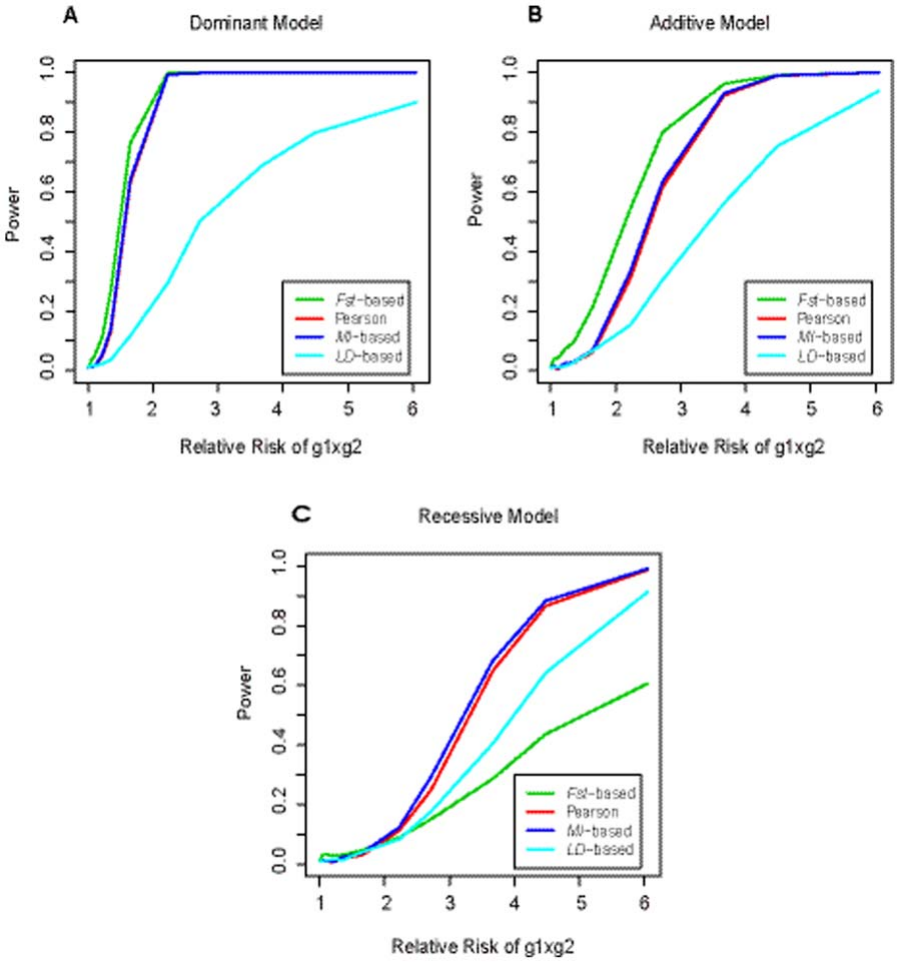
Power of four statistics under three different models when the disease allele frequencies at the two loci are high. The disease prevalence is assumed to be 1%. The disease allele frequencies at the two loci (g1 and g2) are 0.3 and 0.8, respectively. The power, at significance level α of 0.05, is obtained based on simulations of 500 cases and 500 controls. The green, red, blue, and cyan lines are the power of *Fst* based statistic, Pearson's Chi-square statistic, *MI* based statistic, and *LD* based statistic, respectively. Three plots (A, B, C) correspond to the dominant model, the additive model and the recessive model, respectively.

**Figure 5:**
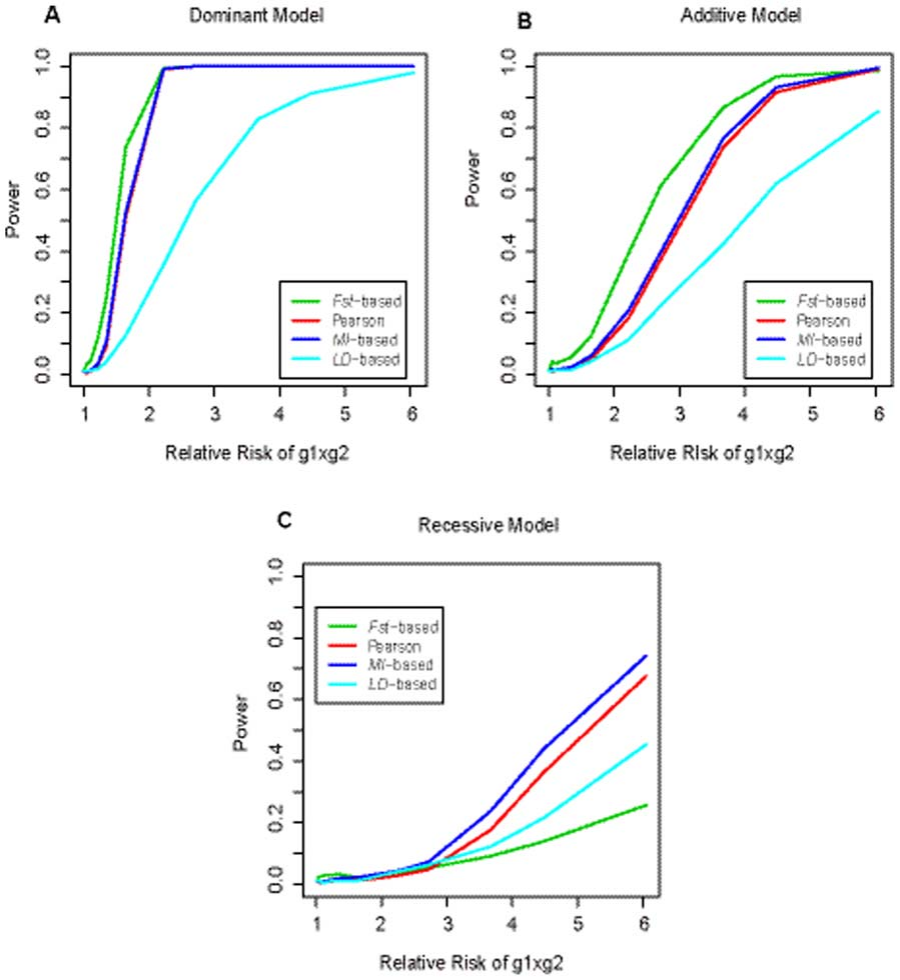
Power of four statistics under three different model when the disease allele frequencies at the two loci are low. The disease prevalence is assumed to be 1%. The disease allele frequencies at the two loci are 0.2 and 0.4, respectively. The power, at significance level α of 0.05, is obtained based on simulations of 500 cases and 500 controls. The green, red, blue, and cyan lines are the power of *Fst* based statistic, Pearson's Chi-square statistic, *MI* based statistic, and *LD* based statistic, respectively. Three plots (A, B, C) correspond to the dominant model, the additive model and the recessive model, respectively.

Figure 4 gives the power curves for the four different methods (*Fst*, Pearson chi-square test, *MI* and *r^2^*). Figure 4a, 4b and 4c corresponds to three interaction models, with disease allele frequencies of 0.3 and 0.8 for the two loci. The three plots (5a, b, c) in Figure 5 give the power curves for the four methods, under three interaction models, but with lower disease allele frequencies (0.2 and 0.4, respectively for the two loci). Obviously, all the curves are climbing as the interaction effect increases. The climbing speed is most fast in dominant model and followed by additive and recessive model in order, indicating that dominant×dominant interactions were more easily to be detected. Both figures show that the *Fst* based method outperformed the others in dominant and additive models no matter what disease allele frequencies were. But, in recessive models, the power of *Fst* was lower than the other three methods when the interaction effect (the relative risk of g1×g2) was large than 2. The *MI* based test seems to have the highest power in recessive models, especially when disease allele frequencies were small. Generally, the *MI* based test had similar power to Pearson's chi-square test, under all interaction models. The correlation between the power values for the two methods was 0.992.

**Figure 6:**
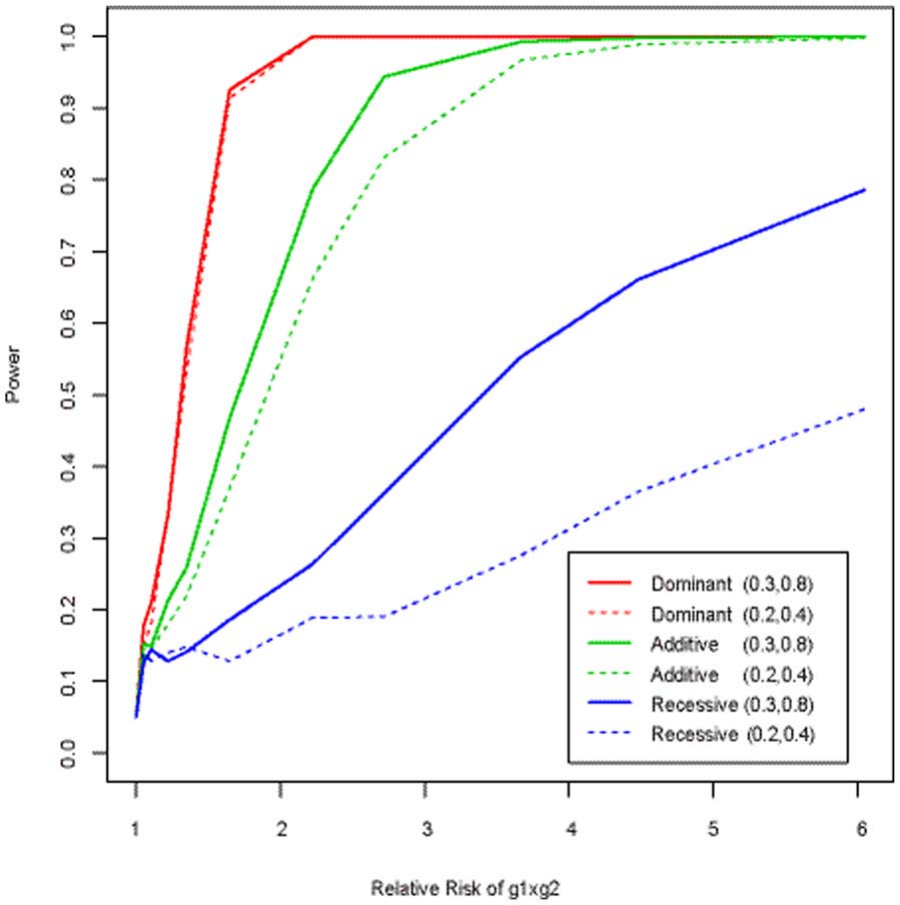
Power of *Fst* under different parameter settings. The disease prevalence is assumed to be 1%. The power, at significance level α of 0.05, is obtained based on simulations of 500 cases and 500 controls. Three solid colorful lines (red, green and blue) correspond to the power curves of the *Fst-*based statistic under three genetic models (dominant, additive and recessive), when the disease allele frequencies at the two loci (g1 and g2) are 0.3 and 0.8, respectively. The dotted lines are the power curves under the assumption that the disease allele frequencies at the two loci are 0.2 and 0.4, respectively.

In order to explore widely the behaviors and performance of the proposed evolution based method, varieties of parameter settings were simulated. The power results are shown in Figure 6, which indicate that the power of *Fst* for detecting gene-gene interaction appears to be not affected by disease allele frequencies under the dominant model. However, in additive and recessive models, the *Fst* based test achieved higher power when the disease allele frequencies were higher. Again, it is evident from this independent simulation experiment that the power of *Fst* was depended on the interaction models: the highest power was achieved in dominant models, followed by additive models. The power for recessive models was not only the lowest, but also strongly affected by the disease allele frequencies.

### Application to a Real Data Example

To further evaluate the performance of *Fst* for detecting gene-gene interaction, a real data example was analyzed. The dataset is a birth cohort study of the incidence of hospital admission with malaria and severe malaria from Kilifi District Hospital on the coast of Kenya in Africa [30]. There were 2104 children in that study, and each was genotyped at both hemoglobin (Hb) and α+- thalassemia genes. The Hb gene had two alleles, denoted as A and S. Allele S was the mutant allele which causes sickle cell disease and A was the normal allele. People with two copies of sickle cell gene suffer terrible pain and die young. So, there was no child with two copies of S in that dataset. Similarly, the gene α+- thalassemia also had the normal and mutant alleles, denoted by α and -, respectively. The proposed *Fst* based method is applied to test the interaction between Hb and α+- thalassemia genes. The results are summarized in Table 1. For comparison, Table 1 also listed the *P*-values obtained by Poisson regression analysis, performed by Williams et al. [30], and the *P*-values obtained by using LD based test in Zhao et al. [28]. The comparison showed that P values of the *Fst* based test were smaller than those of Poisson regression analysis or slightly smaller than those of LD based test. It appears that the *Fst* based test achieved comparable performance to the LD based test.

**Table 1:** Comparison of P-Values for detecting gene-gene interaction.

	Genotypes	No. of cases	No. of controls	P-values obtained by		
				Wald test^1^	*LD*-based test^2^	*Fst* based test
Malaria Admission	*HbAA* & *αα*/*αα*	168	458	0.026	1.4e-5	7.74e-10
	*HbAA* & *-α*/*αα*	187	680			
	*HbAA* & *-α*/*-α*	56	246			
	*HbAs* & *αα*/*αα*	6	107			
	*HbAs* & *-α*/*αα*	9	141			
	*HbAs* & *-α*/*-α*	10	36			
Severe Malaria	*HbAA* & *αα*/*αα*	67	559	0.0012	5.6e-4	2.58e-4
	*HbAA* & *-α*/*αα*	53	814			
	*HbAA* & *-α*/*-α*	17	285			
	*HbAs* & *αα*/*αα*	0	113			
	*HbAs* & *-α*/*αα*	2	148			
	*HbAs* & *-α*/*-α*	5	41			

1 P-values reported by Williams et al.

2 P-values reported by Zhao et al.

## Discussion

In this study, we have proposed a new perspective and a new method to explore gene-gene interactions involved in human disease. Extensive studies have been dedicated to this issue in large-scale association studies, however the distinction and isolation between statistical interaction and biological interaction becomes a major bottleneck in practice [7], [31]. It appears to be of a central role that developing metrics to quantify and confer biological plausibility to the interaction. Therefore, the topic addressed by this article is very insightful and yet not widely exploited. As the prominent geneticist Theodosius Dobzhansky had said: “Nothing in biology makes sense except in the light of evolution”. Our study demonstrates reversely that the evolution concepts and principles can help us analyze the current human population, the outcome from long-time evolution, and recognize genetic variants responsible for phenotypic diversity of human disease. The diversity could be described as the consequence of the long-time evolution process where natural selection of these ancient mutations have occurred in generations. Variations in gamete or haplotype frequencies at multiple loci within the same disease phenotype and among different phenotypes could be interpreted as the magnitude of genetic diversity underling human disease. Alternatively, the gene-gene interplays could reasonably be assumed as the outcome of natural selection of gametes (haplotype) that maximize allele combinations for reproductive success.

From the above perspective, the proposed evolution based method enjoys several merits. It not only directs us to find more informative and more powerful measure to detect genes or gene-gene interactions, proving undetectable by current single-locus methodology, but also help us to trace the origins and progressions of human disease. In the past, evolution science concerns mainly on morphological or physiological characters that have been extensively used for inference of within-species or inter-species evolution. Nowadays, we are glad to see increasing application of modern evolutionary theory to understand health and disease. In nature, many hereditary disease traits are nothing different from morphological or physiological characters, and they all are quantitative traits with complex genetic basis including polygenic background, major genes, and complex gene-gene interplays. Thus, the choice of *Fst*, an evolution concept, as a measure to capture disease evolution, is reasonable.

Sewall Wright [18] and Gustave Malécot [32] introduced *F*-statistics as a tool for describing the partitioning of genetic diversity within and among populations. *Fst*, one of these *F*-statistics, is directly related to the variance in allele frequency among populations and, conversely, to the degree of resemblance among individuals within populations. *Fst* takes a central role in population and evolutionary genetics and has wide applications in fields of disease association mapping. But, to our best knowledge, our study is the first attempt to use the evolution concept for detecting gene-gene interaction. Through large number of simulations and an application to a real example, we find that *Fst* measure is both informative and powerful for detecting gene-gene interactions.

Our results show that the *Fst* based method outperforms several alternative methods in dominant and additive models, no matter what disease allele frequencies are. However, it appears not performing so well in recessive models. This discrepancy might be due to the following reasons. First, for two disease loci, there are nine genotypes: *AABB*, *AaBB*, *aaBB*, *AABb*, *AaBb*, *aaBb*, *AAbb*, *Aabb*, and *aabb*, assuming that *A* and *B* are the disease alleles. In a recessive model, only one of these genotypes, *AABB*, has an epistatic effect according to our genetic coding. And the genotype *AABB* can only create unique gamete *AB*. In dominant or additive models, five genotypes (*AABB*, *AABb*, *AaBB* and *AaBb*) have an epistatic effect. Therefore, in dominant and additive modes, more haplotypes contribute to gene-gene interaction. Because *Fst* is directly related to the variance in gamete frequencies among populations, it performs poorly due to the reduced variance. Second, if a disease is in recessive inheritance, its prevalence is often smaller than a disease with dominant and additive inheritance. From an evolution point of view, the larger the disease prevalence is, the fast the disease evolves, causing more genetically differentiated between the disease population and the health population, which is why the power of the *Fst* based test is higher in dominant and additive models than recessive models. The second reason also explain why *Fst* performs better when disease allele frequencies is higher.

Finally, we should recognize that the *Fst* based method is model-free in nature, and it cannot tell how the genes at the two loci are interacted. Therefore, once meaningful interaction is identified by this method, a model based method has to be used to figure out the best interaction model. Furthermore, this study only investigated the behaviors of the *Fst* based test under three common interaction models, and it remains unclear regarding its capability under other interaction models. Gene-gene interactions might be much more sophisticated than we could image. Hallgrimsdottir and Yuster [33] pointed out that there were 387 distinct types of two locus models, which could reduced to 69 when symmetry between loci and alleles was accounted for. In the future studies, we will aim at exploring the utilities of the proposed methods and alternative approaches to detecting different forms of gene-gene interaction. Finally, to completely decipher the underling genetic interplays for complex diseases, methods for analysis of high-order interactions between multiple loci have to be developed. Although the proposed *Fst* based test can be straightforwardly extended for detecting high-order interactions, the key issue for finding SNP barcodes of genotypes to predict disease susceptibility [34], it remains to be a challenging task computationally.

## Supporting Information

Text S1 Appendix: To prove that the covariance matrix for multivariate discrete sampling approximates asymptotically the one for multivariate normal sampling.
(DOC)

Figure S1 Comparison of the elements in two sampling's covariance matrices.
(TIF)
