# Supplementary material for: A Novel Evolution-Based Method for Detecting Gene-Gene Interactions
Source: PLoS One. 2011 Oct 25;6(10):e26435. doi: 10.1371/journal.pone.0026435 (PMC3201950; doi:10.1371/journal.pone.0026435)
Supplement: Text S1 — Appendix: To prove that the covariance matrix for multivariate discrete sampling approximates asymptotically the one for multivariate normal sampling. (DOC) [file pone.0026435.s001.doc]

**Appendix: To prove that the covariance matrix for** **multivariate discrete sampling approximates asymptotically the one for multivariate normal sampling**

**1 Proof**

By definition,$\mathbf{x}_{n}=(x_{n}^{(A)},x_{n}^{(B)})$, *n*=1,2,3,…, is the *n*th sample at the two disease loci. Consider the following covariance matrix:

.

It can be decomposed into two parts by adding and subtracting *μ_A_* or *μ_B_*

$$=\frac{1}{n}\left(\begin{matrix} \sum_{i=1}^{n} (x_{i}^{A}-\mu_{A})^{2} & \sum_{i=1}^{n} (x_{i}^{A}-\mu_{A})(x_{i}^{B}-\mu_{B}) \\ \sum_{i=1}^{n} (x_{i}^{A}-\mu_{A})(x_{i}^{B}-\mu_{B}) & \sum_{i=1}^{n} (x_{i}^{B}-\mu_{B})^{2} \end{matrix}\right)-$$

For the second part of the above decomposition, according to the law of large number, we have

By the property of convergence in probability, we have

Therefore, the second part of the above decomposition converges to zero in probability. Note that the first decomposition can be written as the sum of *n* i.i.d random matrix:

$\frac{1}{n}\sum_{i=1}^{n} \left(\begin{matrix} (x_{i}^{A}-\mu_{A})^{2} & (x_{i}^{A}-\mu_{A})(x_{i}^{B}-\mu_{B}) \\ (x_{i}^{A}-\mu_{A})(x_{i}^{B}-\mu_{B}) & (x_{i}^{B}-\mu_{B})^{2} \end{matrix}\right)$.

According to the law of large number, we have

，

where $h_{AB}$ is the probability of genotype *AB*. Therefore, we have

According to the *slutsky* theorem, the summation of the above decomposition converges to the following matrix

$\left(\begin{matrix} \mu_{A}(1-\mu_{A}) & h_{AB}-\mu_{A}\mu_{B} \\ h_{AB}-\mu_{A}\mu_{B} & \mu_{B}(1-\mu_{B}) \end{matrix}\right)$，

that is to say, the covariance matrix of $X=\left(X^{\left(A\right)},X^{\left(B\right)}\right)^{T}$ converges to the above matrix in probability, i.e.

, when $n\to\infty$.

**2 Simulation**

To make the above proof visualized, we did extensive simulations as following: (1) we randomly generated the multinomial parameter **h**=(*h_AB_*, *h_Ab_*, *h_aB_*, *h_ab_*). Based on this parameter, we generated *n* *multinomial* (1, **h**) samples and translated them to two dimension random vector ***X***. Based on this **h**, *n* multivariate normal distribution,

$N_{2}\left[\left(\begin{matrix} \mu_{A} \\ \mu_{B} \end{matrix}\right),\left(\begin{matrix} \mu_{A}(1-\mu_{A}) & h_{AB}-\mu_{A}\mu_{B} \\ h_{AB}-\mu_{A}\mu_{B} & \mu_{B}(1-\mu_{B}) \end{matrix}\right)\right]$, where $\mu_{A}=h_{AB}+h_{Ab},\mu_{B}=h_{AB}+h_{aB}$,

random vectors were generated; (2) we then calculated the covariance matrixes of the two kinds of sampling. We repeated it 1000 times and thereby 1000 pairs of covariance matrixes were obtained. Finally we compared each corresponding elements of these matrixes, and showed their scatter plots in Figure S1. The first panel shows the scatter plots of the *X*_A_’s variance obtained by two kinds of sampling, and from left to right, *n*=100, 500, 1000 and 5000. The second panel shows the covariance of *X_A_* and *X_B_* under different sample size *n*. The third panel is for the variance of *X_B_* similar to the first panel. It can be seen that each element in the covariance matrix of X, obtained by the multivariate discrete sampling, converges to the correspond elements, obtained by the multivariate normal sampling.

**
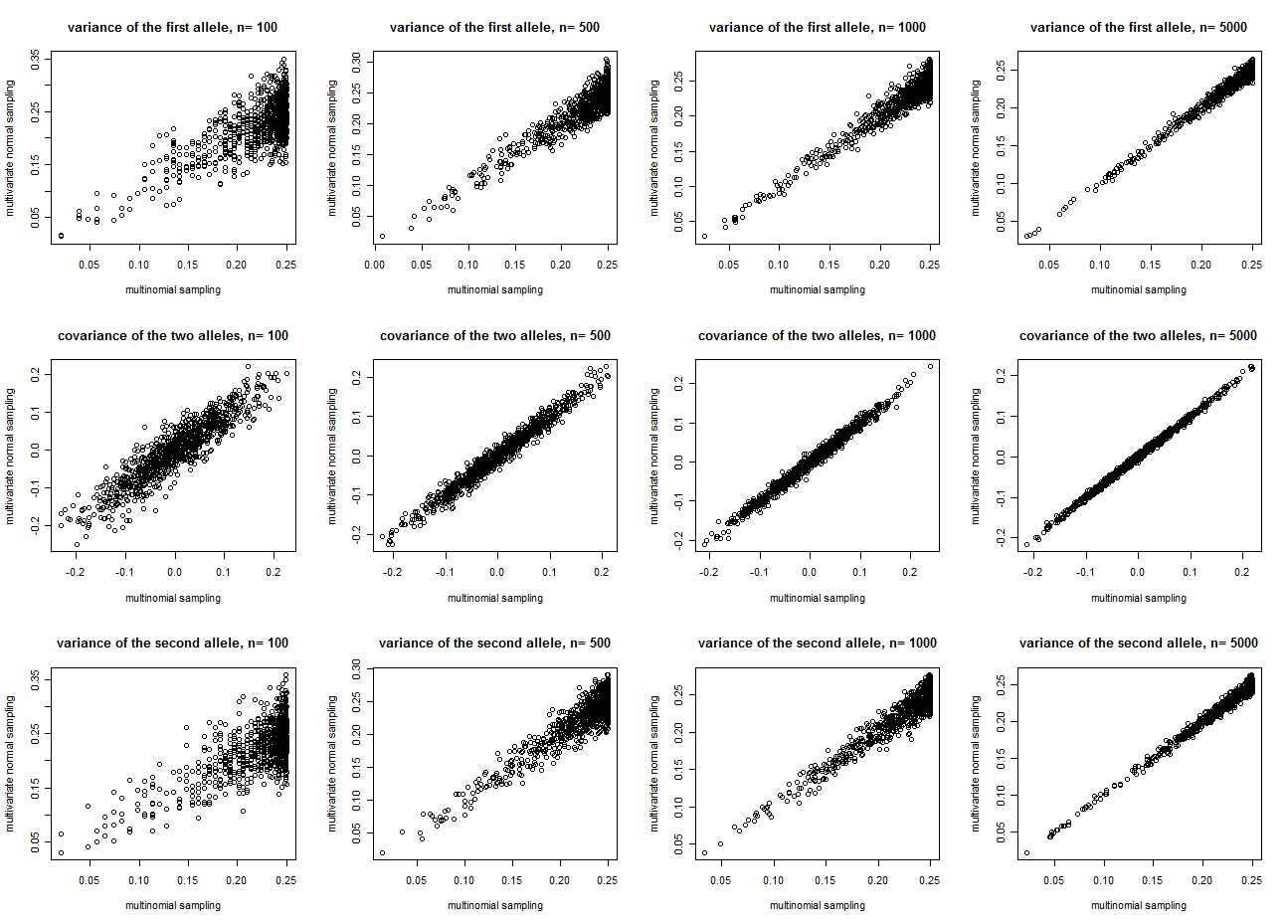
**

**Figure S1. Comparison of the elements in two sampling’s covariance matrices.** The plots show the scatter plots of the 3 elements (two allelic variances and the covariance between two loci) in the covariance matrices under two samplings. The middle panel shows the covariance of two variables, and the others show the allelic variances at the two loci, respectively. From left to right, the sample size is increasing: 100,500, 1000 and 5000, respectively.
