# Supplementary figures and images for: A Novel Evolution-Based Method for Detecting Gene-Gene Interactions
Source: PLoS One. 2011 Oct 25;6(10):e26435. doi: 10.1371/journal.pone.0026435 (PMC3201950; doi:10.1371/journal.pone.0026435)

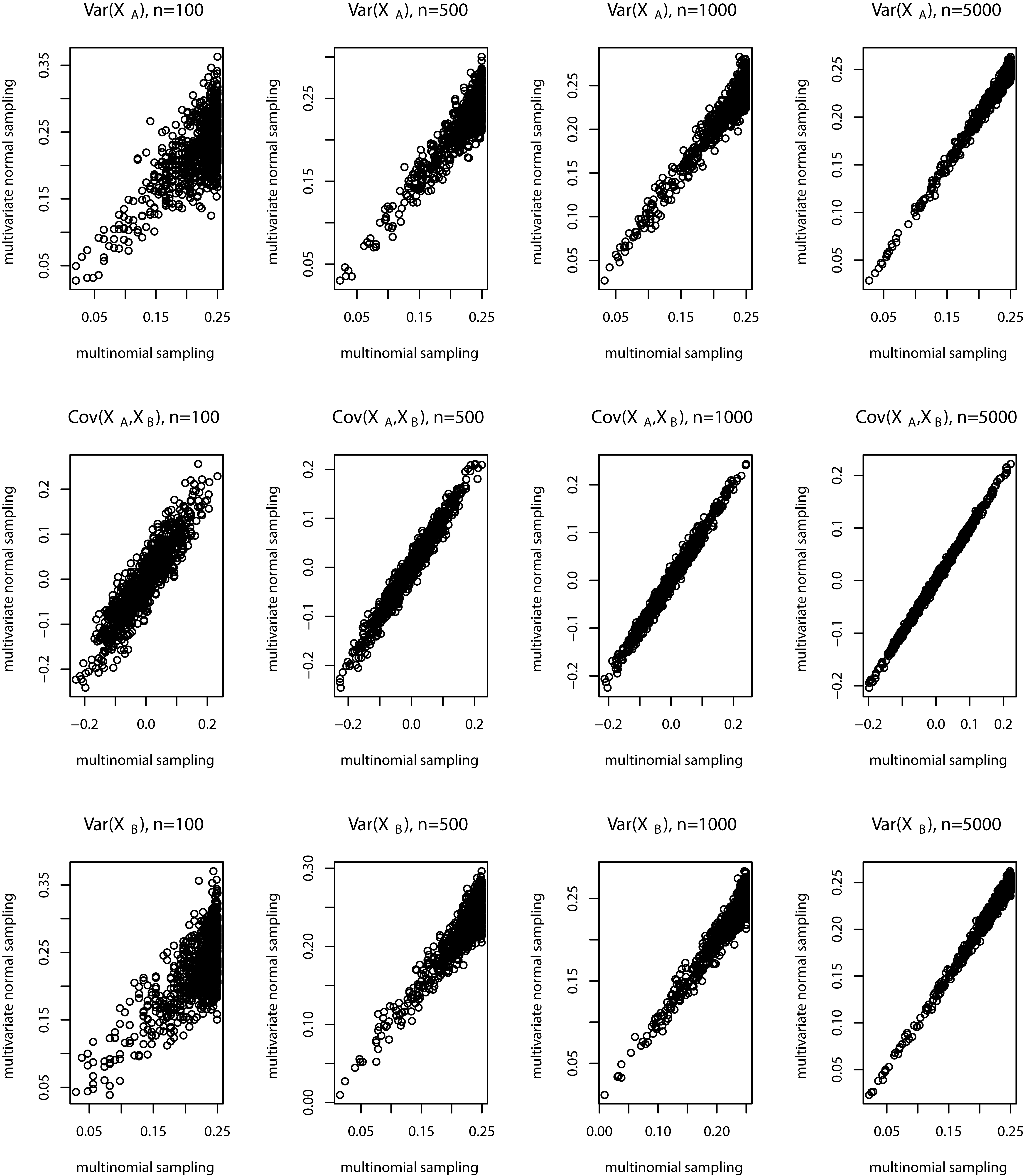

Supplement: Figure S1 — Comparison of the elements in two sampling's covariance matrices. (TIF) [file pone.0026435.s002.tif]
